# Bodily awareness: Religious culture’s associations with interoceptive sensibility

**DOI:** 10.1371/journal.pone.0309216

**Published:** 2024-12-02

**Authors:** Patty Van Cappellen, Tehya M. LePage Drummond

**Affiliations:** 1 Social Science Research Institute, Duke University, Durham, North Carolina, United States of America; 2 Department of Psychology and Neuroscience, University of North Carolina at Chapel Hill, Chapel Hill, North Carolina, United States of America; Federal University of Rio de Janeiro: Universidade Federal do Rio de Janeiro, BRAZIL

## Abstract

Religions, as cultural systems, influence how people view and attune to their body. This research explores whether individual differences in various dimensions of religiosity are associated with interoceptive sensibility (IS), i.e., one’s perceived ability to detect and interpret bodily signals. In Study 1, Christians, Muslims, and Hindus (*N* = 1570) reported their religiosity and completed the Multidimensional Assessment of Interoceptive Awareness, a well-validated measure of IS. Results show that religious identity moderates the relationship between the centrality of religion in one’s life and IS such that the association is positive and medium for Christians, large for Muslims and Hindus. In addition, the medium positive correlation between frequency of religious practice and IS was similar across religious groups. Study 2 (*N* = 450) extended these results by measuring additional dimensions of religiosity and spirituality as well as investigating religious-related beliefs about the body, both positive (e.g., My body is holy) and negative (e.g., My body is sinful). Associations between religiosity and IS are replicated and found for spirituality as well. Interestingly, mediation analyses reveal that belief in the body as holy partially explains the association between religiosity and IS, but belief in the body as sinful suppresses such association. We discuss how religion, as a cultural factor, may influence beliefs about the body and bodily awareness, with implications for emotion regulation and mental health.

## Introduction

Humans’ nuanced awareness of the body’s internal signals plays a critical role in shaping our emotional landscape and overall well-being [1,2]. Yet, the extent to which such perception is shaped by cultural factors such as religious beliefs and practices remains largely uncharted territory [3]. Our research aims to shed light on the nexus between religion and the perception of bodily signals.

Interoception, the brain and mind’s representation of physiological signals, comprises several distinct facets [4–6]. Two major facets of interoception are *interoceptive accuracy*, sometimes referred to as interoceptive sensitivity, and *interoceptive sensibility* (IS). Interoceptive accuracy is measured behaviorally and pertains to one’s objective ability to precisely detect bodily signals (e.g., heartbeat) while IS, often assessed through self-report questionnaires, refers to an individual’s perceived ability to detect, interpret, or regulate bodily sensations and physiological processes such as heartbeat, breath, and emotional arousal [5–9]. Prior research has established that interoceptive accuracy and IS are not necessarily correlated and often predict different outcomes. Notably, IS is distinctly important for assessing conscious physiological experiences and has been shown to independently predict various affective and emotional processes [10–12]. In addition, researchers have suggested that IS is more driven by cultural schemas [e.g., 13], whereas accuracy is more driven by bodily cues [e.g., 14].

For the purposes of our study, we will study IS as indexed by the Multidimensional Assessment of Interoceptive Awareness, [MAIA; 15,16] a well-validated self-report questionnaire of IS. The MAIA comprises eights subscales that tap into different dimensions of IS [15]. Five of the eight MAIA subscales measure general physiological sensibility (i.e., Noticing, Emotional Awareness, Attention Regulation, Body Listening, and Trusting) and three subscales measure either adaptive or maladaptive regulatory strategies (i.e., Not-Distracting, Not-Worrying, and Self-Regulation) [17]. Although many studies have examined the MAIA on a multidimensional level (i.e., analyzing the subscales independently), researchers have identified a general index of self-reported interoception from the MAIA, excluding the Not-Worrying and Not-Distracting subscales [18,19]. Thus, building upon these insights [18,19], our study will evaluate a general IS index using the five MAIA subscales that measure general physiological sensibility [17]. We also explore each subscale separately, which are presented in the Supporting Information file.

Beyond the physiological domain, IS plays a pivotal role in emotional experiencing, mental health, and overall well-being [6,11,20]. Research has established that several dimensions of IS have been linked to greater emotion regulation [11,21,22] and lower alexithymia, difficulties in identifying and describing emotions [12,22]. Further, dimensions of IS are negatively related to mental health symptoms such as depression and predict psychological improvement trajectories [23–25].

Given this role of IS, researchers have sought to understand the factors that promote it [26]. The present research focuses on cultural factors associated with perceptions of the body. In particular, we study religion: an aspect of culture because it involves a socially-constructed and -transmitted set of beliefs, norms, ritualized practices, and communities [27]. Religion is certainly widespread; in 2015, approximately 84% of the world’s population, or 6.12 billion people, identified with a religion. This figure is projected to grow by 32% by 2060, reaching 8.41 billion religious individuals. Christians, Muslims, and Hindus are the main contributors to this growth [28].

Religious beliefs and rituals offer a unique lens through which the body is often viewed and experienced. Specific contemplative practices embedded in various religious traditions, such as meditation and mindfulness in Buddhism, focus on bodily awareness, thereby enhancing sensitivity to internal states [3,29]. Ritualistic practices such as prayer and worship involve specific physical postures and draw attention to how the body is positioned [30–32]. Furthermore, ascetic practices, including fasting, inherently heighten attention to bodily needs and responses, potentially amplifying interoceptive awareness [33]. One study found that fasting (outside of a religious context) led to more interoceptive accuracy [34]. Finally, traditions like Hinduism emphasize the interconnection of mind, body, and spirit to promote a holistic awareness of bodily sensations [35]. Empirical research has focused on testing whether specific embodied contemplative practices can improve IS. Overall, people who engage in mind-body practices such as yoga, mindfulness meditation, or body scan meditation show higher IS [e.g., 36–38] but notably not interoceptive accuracy [39,40]. However, no research to our knowledge has investigated whether engaging in traditional religious practices such as prayer and worship is associated with IS.

Religiosity refers to the individual difference in being interested in and/or involved with religion [41]. Much research in sociology and psychology differentiates between three indicators of religiosity [42]: (1) *centrality of religion* refers to a global evaluation of the extent to which religion is important or central in person’s life [43], (2) *religious practice* refers to the frequency of participation in collective and private religious practices such as attending religious services or individual prayer, and (3) *affiliation* with a particular religious tradition. To these dimensions we add modern spirituality, which has received much scientific attention in the past 25 years. *Spirituality* refers to the search for or connection with what is perceived as sacred [44]. Spirituality can be experienced within a religion–tapping into more emotional aspects of religion–or outside a religious framework.

Recognizing that religious traditions may differ on their teachings and ways to relate to the body, we consider the impact of religiosity dimensions and of ascribing to specific views of the nature of the body. Regarding the latter, many traditions rely on a dualistic view of the body, recognizing its importance and the need for care, while also often placing greater emphasis on the spiritual aspect of human existence. Focusing on Christianity, two very different theologies around the body co-exist [45,46]. One emphasizes the body as a temple of the Holy Spirit, to be cared for and respected [47]. Another views the body more negatively, associating it with sin or temptation. Such religiously-based views on the body may further play a role in how religion impacts IS. Relatedly, extensive research has examined the relationship between interoception and body image, consistently showing that more positive body image and body satisfaction is associated with higher IS [48–51].

Despite the intuitive influences of religion on interoception, to our knowledge, no research has empirically examined the relationship between individual differences in religiosity and IS. We aim to fill this gap and measure various dimensions of religiosity.

Our initial study aims to assess whether religiosity correlates with IS among a large sample of U.S. adults who identify as Christians, Muslims, and Hindus. We explore whether such association differs by religious traditions (Study 1). We further explore various dimensions of religiosity, distinguishing between centrality of religion–how central or important religion is in someone’s life–and frequency of religious practices (Studies 1–2). Finally, in Study 2, we explore additional dimensions of religiosity, including spirituality, and evaluate the role of positive and negative religiously-based views of the body (body as holy and body as sinful) for the relationship between religiosity and IS. We reasoned that religion may promote both views [46], but depending on which view someone holds, they may be differently attuned to their body sensations. Together, this research aims to contribute to our understanding of religious/cultural factors that are associated with greater IS.

## Study 1

In Study 1 we explored the associations between religiosity measures (i.e., centrality of religion in one’s life and frequency of religious practices) and IS, and whether such associations differ by religious affiliation (i.e., Christians, Muslims, and Hindus). We further explored which dimension of religiosity was most strongly associated with IS. We use an archival dataset to test these questions (preregistration available here: https://osf.io/2fxjg, see also 32).

## Materials and methods

### Participants

Procedure and materials were approved by the Institutional Review Board at the institution of the first author. As approved by the IRB, participants gave consent electronically prior to participating in the research. As part of a larger study that ran February-May 2019, participants responded to questions about their religious postures, emotions, and personality, in addition to questions about their IS and religious beliefs and practices. Participants were recruited through Amazon’s Mechanical Turk and Turk Prime. To participate, interested individuals needed to identify as Christian, Muslim, or Hindu and live in the U.S. The study was advertised as a 45-minute psychology survey on postures and emotions across religions. In order to achieve our desired sample size for each group, which we preregistered to be 750 Christians, 500 Muslims, and 500 Hindus, our advertisements were targeted toward each religious group individually. Participants were compensated $3.00 USD after completing the survey online. Those who did not meet our inclusion requirements or pass attention check questions were eliminated from participation and were not compensated. After data collection was complete, out of the 1707 participants, a total of 193 participants were excluded from the analyses due to the following criteria: they finished the survey in less than five minutes or failed to follow directions to written response questions. The final set of participants (*N* = 1570; Christian *n* = 674; Muslim *n* = 494; Hindu *n* = 402) comprised 47.1% female, 52.7% male, and 0.1% other. Ages ranged from 18–81 years (*M* = 35.2, *SD* = 11.7). Participants identified as White (48.7%), Asian (30.4%), African American (11.2%), American Indian or Alaska Native (4.3%), other (4.5%), and Native Hawaiian or other Pacific Islander (0.6%).

### Measures

#### Centrality of religion

To assess centrality of religion, we used a three-item measure developed by Ladd [52,53] and following Markus [54] which taps into the centrality of religion in the minds of the participants. Participants rated the extent to which they agreed with each item on a scale from 0 (*not at all*) to 6 (*completely*). The items they answered were as follows: “To what extent do you consider yourself a religious person?”, “To what extent is your stance on religious issues important to you?”, and “To what extent is it likely that other people may view you as a very religious individual?”. Reliability for this scale was high, Cronbach’s α = .84.

#### Frequency of religious practices

To assess the frequency of religious practices, we used two items from Idler and colleagues [55]. Participants were asked “How often do you attend religious services?” and rated their attendance on a scale from 0 (*never*) to 8 (*several times a week*) and “How often do you pray privately in places other than at a specific religiously oriented building?” and rated their prayer frequency on a scale from 0 (*never*) to 7 (*more than once a day*). Responses were Z-scored and then averaged. Reliability for this scale was satisfactory, as indexed by Spearman’s *r* = .35, *p* < .0011, which is recommended for reliability tests of two-item scales [56].

**IS.** To assess IS, the Multidimensional Assessment of Interoceptive Awareness, Version 1 (MAIA; 15) was used. In the larger study from which we draw, only the five subscales that most directly tap into IS (as in 17) were administered and were averaged to create a general IS score (Cronbach’s α = .93): Noticing (α = .66; 3-items; e.g., “When I am tense I notice where the tension is located in my body”), Attention Regulation (α = .86; 7-items; e.g., “I can pay attention to my breath without being distracted by things happening around me”), Emotional Awareness (α = .79; 5-items; e.g., “I notice how my body changes when I am angry”), Body Listening (α = .74; 3-items; e.g., “I listen for information from my body about my emotional state”), and Trusting (α = .72; 3-items; e.g., “I trust my body sensations”). Participants rated each item on a scale from 1 (*never*) to 5 (*always*).

## Results

Analyses were performed on Jamovi version 2.3.21.0 (retrieved from https://www.jamovi.org). Missing data were left as missing and not imputed. Means and standard deviations for all the measures, for the whole sample and by religious identity, are provided in Table 1. Correlations for the whole sample and by religious identity are provided in Table 2. More detailed tables containing all MAIA subscales are provided in the (S1 and S2 Tables).

**Table 1:** Study 1 and 2 means and standard deviations.

	Study 1								Study 2	
	All religions		Christians		Muslims		Hindus			
	*M*	*SD*	*M*	*SD*	*M*	*SD*	*M*	*SD*	*M*	*SD*
IS	3.37	.67	3.33	.73	3.42	.65	3.34	.56	3.30	.84
Religious Centrality	4.41	1.28	4.24	1.42	4.57	1.20	4.49	1.09	3.75	3.11
Frequency of Religious Practice	0	.82	-.23	.88	.27	.73	.05	.69	0	.89
4 Dimensions of Religiousness	N/A	N/A	N/A	N/A	N/A	N/A	N/A	N/A	3.04	2.00
Belonging	N/A	N/A	N/A	N/A	N/A	N/A	N/A	N/A	2.84	2.07
Behaving	N/A	N/A	N/A	N/A	N/A	N/A	N/A	N/A	2.99	2.23
Believing	N/A	N/A	N/A	N/A	N/A	N/A	N/A	N/A	3.16	2.04
Bonding	N/A	N/A	N/A	N/A	N/A	N/A	N/A	N/A	3.15	1.97
Importance of Spirituality	N/A	N/A	N/A	N/A	N/A	N/A	N/A	N/A	4.43	3.21
Daily Spiritual Experiences	N/A	N/A	N/A	N/A	N/A	N/A	N/A	N/A	2.68	1.64
Body As Holy	N/A	N/A	N/A	N/A	N/A	N/A	N/A	N/A	3.34	2.25
Body As Sinful	N/A	N/A	N/A	N/A	N/A	N/A	N/A	N/A	2.16	1.70

**Table 2:** Pearson’s r correlation matrix between Interoceptive Sensibility (IS) and religious measures for all and by religious group (Study 1).

	All		Christians		Muslims		Hindus	
	IS	CR	IS	CR	IS	CR	IS	CR
Religious Centrality (CR)	.30***	—	.20 ***	—	.37***	—	.48***	—
Frequency of Religious Practice	.17***	.57***	.13**	.66***	.17***	.50***	.23***	.39***

Note

** p < .01

*** p < .001.

First, we tested whether religious affiliation moderated the associations between our indicators of religiosity (independent variable) and IS (dependent variable) using a set of linear regressions. Religious affiliation moderated the association between centrality of religion and IS, *F*(2, 1557) = 10.7, *p* < .001. Therefore, Pearson’s correlations (see Table 2) between the two constructs were computed for each of the three religions individually. As expected, variables were not normally distributed, but using Spearman’s rank correlation led to similar findings as the Pearson’s correlation. Results showed that the positive association between the two constructs was medium for Christians and large for Muslims and Hindus [57]. Next, we conducted Fisher’s Z transformation tests to determine whether correlations differed significantly between the three groups. Results showed that the positive association between the two constructs was smaller for Christians compared to Muslims and Hindus (respectively, *z* = 3.13, *p* < .01; *z* = 5.07, *p* < .001), but Muslims and Hindus barely differed from each other (*z* = 2, *p* = .046).

The same moderation tests were conducted for frequency of religious practices, but the linear regression revealed no significant interaction between frequency of religious practice and IS by religious affiliation (*F*(2, 1557) = 1.24, *p* = .290). Therefore, we computed a correlation for the entire sample. We found a statistically significant, small to medium positive correlation between frequency of religious practice and IS, *r*(1563) = .17, *p* < .001.

Finally, in the entire sample, using Fisher’s Z transformation to test whether IS is more strongly associated with centrality of religion or with frequency of religious practices, we found that the correlations statistically differed from each other (*z* = -3.85, *p* < .001), showing that IS is more strongly associated with centrality of religion.

In sum, indicators of religiosity are *positively* associated with IS. Such association is stronger for the centrality of religion than for frequency of religious practice. We further note that the associations between centrality of religion and IS differed by religious traditions, weakest among Christians and strongest among Muslims and Hindus.

## Study 2

Study 2 aimed to replicate and extend Study 1 and was preregistered on aspredicted.org (#124909). We aimed to conceptually replicate the test for the associations between religiosity measures and IS. We extended Study 1 by including a multidimensional measure of religiosity and measures of spirituality and exploring their associations with IS. Regarding religiosity, we included a validated measure of four major religion dimensions as identified by Saroglou and colleagues [58], known as the "four Bs": Believing (specific ideas about the transcendent and its relation to humans and the world), Bonding (emotional connections through private or collective rituals with the transcendent), Behaving (conforming to norms, practices, and values perceived as established by the transcendent), and Belonging (association with a group perceived as eternal and filled with the transcendent presence) [58]. Regarding spirituality, we measured the centrality of spirituality in one’s life and frequency of spiritual experiences in daily life.

We preregistered the following hypotheses and research questions (edited for clarity): We hypothesize a small, positive correlation between centrality of religiosity and IS, and a smaller but positive correlation between frequency of religious practices and IS. We will explore the association between IS and other facets of religiosity/spirituality (4 facets of religiousness, 58, and daily spiritual experiences, 59). We will also explore whether religious affiliation (if we have groups large enough for Muslims or Hindus) moderates the association between religiosity and IS.

We further aimed to explore the role of religious-based beliefs about the body–as sinful and as holy–on the association between religiosity and IS. We preregistered a moderation: We will also explore whether views about the body (sinful and holy) moderate the association between religiosity and IS. We expect that religiosity’s association with IS will be reduced or even negative among people who have a more sinful view of the body, but will be more positive among people who have a more sanctified view of the body.

Finally, we preregistered to also explore associations between views about the body and the MAIA subscales. Based on Jacobson and colleagues [46] and Todd and colleagues [51], we expect that the Noticing subscale will be associated with a more sinful view of the body, and the Attention Regulation, Self-Regulation, and Trusting subscales will be associated with a more sanctified view of the body.

## Method

### Procedure

Procedure and materials were approved by the Institutional Review Board at the institution of the first author. As approved by the IRB, participants gave consent electronically prior to participating in the research. As part of a larger study run in March 2023, participants responded to questions on IS, religiosity, and beliefs about their body. We collected these measures at the very end of a survey testing the association between religiosity/spirituality and compassion collapse. Participants were randomly assigned to see one picture of a child vs. a seven-picture collage of children suffering. We preregistered that we would test whether conditions affected the present measures and that if they did, we would control for conditions. Since there were small differences between conditions on IS (*p* = .035, *d* = .20), Daily Spiritual Experiences (*p* = .024, *d* = .21), and belief in body as holy (*p* = .036, *d* = .20), we controlled for conditions.

### Participants

Participants were recruited through Connect by CloudResearch. To participate, interested individuals needed to be 18 or older, live in the U.S., and speak fluent English. The study was advertised as a study on beliefs and opinions. Participants were compensated $1.30 USD after completing this 10-minute survey online. Those who used a VPN or did not pass attention check questions were eliminated from participation and were not compensated. We set a priori to collect data among 450 participants and collected such sample (*N* = 450, 50.2% female and 49.8% male). Ages ranged from 18–77 years (*M*_age_ = 40.8, *SD*_age_ = 12). For religious affiliation, participants identified as Protestant (22.7%), Agnostic (21.1%), Atheist (18.9%), Catholic (16.9%), spiritual but not religious (12.9%), other (3.6%), Jewish (2.4%), Buddhist (1.4%), and Muslim (.2%). Participants identified as White (81.6%), African American (9.6%), Asian (6.7%), American Indian or Alaska Native (.2%), other (1.6%), and Native Hawaiian or other Pacific Islander (.4%).

### Measures

#### Centrality of religion

This study administered a different measure than in Study 1 as developed by Saroglou and Munoz-Garcia [60], but that still taps into perceptions of centrality of religion in one’s life (see for a discussion, 43). Participants rated the extent to which they agreed with the following statements on a scale of 1 (*Not at all*) to 9 (*Yes*, *extremely*): “How much is God important in your life?” and “How much is religion important in your life?”. Reliability for this scale was high as indexed by Spearman’s *r* = .88, *p* < .001.

#### Frequency of religious practices

Participants reported their frequency of practice in private prayer and collective worship using the same measure as in Study 1 [55]. Reliability for this scale was moderate, with a Spearman’s *r* = .61, *p* < .001.

#### Spirituality

Participants completed two measures to assess centrality of spirituality and frequency of spiritual experiences. First, they rated “How much is spirituality important in your life?” on a scale of 1 (*Not at all*) to 9 (*Yes*, *extremely)* [60]. To assess how spirituality is expressed and experienced in daily life, we used the 6-item Daily Spiritual Experiences Scale [59]. Participants rated how often they may have the following experiences in their daily lives from 1 (*Never to almost never*) to 6 (*Many times a day*). Example items include “I feel God’s presence”, “I feel deep inner peace or harmony”, and “I desire to be closer to or in union with God.” They were informed that they could replace the word “God” with any experiences with the transcendent, the divine, or a Higher Power when answering these questions. Reliability for this scale was high, with a Cronbach’s α = .96.

#### Four dimensions of religiousness

We used The Four Basic Dimensions of Religiousness Scale [58]. On this 12-item scale, participants rated the items on a scale of 1 (*totally disagree*) to 7 (*totally agree*). The scale consists of four subscales: Belonging Subscale (α = .94; e.g., “In religion, I enjoy belonging to a group/community”), Behaving Subscale (α = .97; e.g., “Religion helps me to try to live in a moral way”), Bonding Subscale (α = .92; e.g., “I like religious ceremonies”), and the Believing Subscale (α = .90; e.g., “I feel attached to religion because it helps me to have a purpose in my life”). Reliability for this scale was high, with a Cronbach’s α = .98.

#### IS

To assess IS, we used the Multidimensional Assessment of Interoceptive Awareness, Version 2 (MAIA-2; 16). Following the same rationale as in Study 1, we computed a general IS score (Cronbach’s α = .94) based on five subscales: Noticing (α = .83; 4-items), Attention Regulation (α = .90; 7-items), Emotional Awareness (α = .87; 5-items), Body Listening (α = .88; 3-items), and Trusting (α = .92; 3 items). We also administered the rest of the scale to explore associations with other subscales: Self-Regulation (α = .88; 4 items; e.g., “When I bring awareness to my body I feel a sense of calm.”), Not Distracting (α = .89; 6-items; e.g., “I distract myself from sensations of discomfort.” [reverse scored]), and Not Worrying (α = .78; 5-items; e.g., “I can notice an unpleasant body sensation without worrying about it.”).

#### Religious-related beliefs about the body

To assess positive religious-related beliefs about the body (Body As Holy), we chose four items from the 14-item Christian Teachings on the Body Scale [46] due to time constraints. These items were selected at face value because they strongly represent religious-related positive beliefs about the body: “My body is blessed”, “My body is holy”, “My body is a temple of God”, and “My body is a gift from God.” Participants rated each item on a scale from 1 (*strongly disagree*) to 7 (*strongly agree*). Reliability for this subscale was high, Cronbach’s *α* = .97.

To assess negative beliefs, we again chose at face value four items from the 12-item Radical Dualism Scale [46] that we felt accurately captured negative religious-related beliefs about the body (Body As Sinful). The four chosen items were rated on a scale from 1 (*strongly disagree*) to 7 (*strongly agree*): “My body is just something I live in here on earth”, “My body is basically sinful”, “My soul is more important to God than my body”, and “My body frequently causes me to sin”.

An exploratory factor analysis (EFA) was conducted on all items to identify the underlying factor structure. The Kaiser-Meyer-Olkin measure verified the sampling adequacy for the analysis, KMO = 0.85, and Bartlett’s Test of Sphericity was significant (p < .001), indicating that correlations between items were sufficiently large for EFA. Principal Axis Factoring was used as the extraction method, followed by Promax rotation. The initial analysis revealed two factors with eigenvalues greater than 0.72, all the positive beliefs loaded on one factor (all factor loadings > .80) and two of the negative beliefs (i.e., body as sinful) loaded on the second (all factor loadings > .90). A third factor had an Eigenvalue of only 0.11. This third factor comprised two items: “My body is just something I live in here on earth” (factor loading = .44) “My soul is more important to God than my body” (factor loading = .85). We decided to retain only the items for the two factors with eigenvalues greater than 0.72 and this final model explained 87% of the total variance. Body as holy remained unchanged and body as sinful comprised the two items with the most face validity: “My body is basically sinful,” “My body frequently causes me to sin” (Spearman’s *r* = .82, *p* < .001)

## Results

Analyses were performed on Jamovi version 2.3.21.0. Missing data were left as missing and not imputed. Means and standard deviations for all the measures are provided in Table 1. A full correlation matrix can be found in Table 3. A more detailed correlation matrix with IS subscales is available in the (see S3 Table).

**Table 3:** Correlation matrix between IS, religious measures, and beliefs about the body (Study 2).

	1	2	3	4	5	6	7	8	9	10	11
1. IS	—										
2.Centrality of Religion	.21**	—									—
3. Frequency of Religious Practice	.17***	.83**	—								
4. Importance of Spirituality	.26***	.84***	.69***	—							
5. Daily Spiritual Experience	.32***	.90***	.82***	.83***	—						
6. 4 Dimensions of Religiousness	.22***	.90***	.81***	.76***	.85***	—					
7. Belonging	.22***	.87***	.79***	.73***	.81***	.97***	—				
8. Behaving	.20***	.89***	.79***	.73***	.83***	.97***	.93***	—			
9. Believing	.21***	.89***	.79***	.78***	.85***	.97***	.91***	.93***	—		
10. Bonding	.24***	.80***	.72***	.69***	.77***	.94***	.90***	.86***	.88***	—	
11. Body as Holy	.33***	.85***	.74***	.81***	.88***	.78***	.74***	.76***	.80***	.70***	
12. Body as Sinful	.01	.45***	.41***	.38***	.40***	.39***	.38***	.39***	.38***	.35***	.45***

Note. Analyses control for Condition variable

* *p* < .05

** *p* < .01

*** p < .001.

### Associations between IS and religion/spirituality measures

Supporting our hypothesis, we found a small to medium positive association between IS and centrality of religion as well as with frequency of religious practices [57]. However, unlike in Study 1, the size of these associations did not statistically significantly differ from each other, *z* = -0.62, *p* = .535. Other measures of religiosity and spirituality showed similar associations, with the Daily Spiritual Experience scale being statistically significantly more strongly associated than frequency of religious practices (*z* = 2.39, *p* = .017), but not than other measures.

### Role of beliefs about the body

We first examined whether beliefs about the body moderated the association between religiosity and IS using the medmod module in Jamovi. A significant interaction between centrality of religion and belief in the body as holy on IS emerged, *b* = 0.03, *SE* = .01, *p* < .001. Simple slopes analyses revealed that at low (-1*SD*) and average (*M*) levels of belief in body as holy, the association between centrality of religion and IS was statistically significant but negative (respectively, *b* = -0.17, *SE* = .03, *p* < .001; *b* = -0.10, *SE* = .01, *p* < .001). At high (+1*SD*) levels of belief in the body as holy, such association was only marginally significant, *b* = -0.03, *SE* = .02, *p* = .074. A similar pattern was observed for frequency of religious practices and IS by positive beliefs about the body (significant interaction: *b* = 0.08, *SE* = .03, *p* < .001). At low (-1*SD*) and average (*M*) levels of belief in body as holy, the association between centrality of religion and IS was statistically significant but negative (respectively, *b* = -0.47, *SE* = .10, *p* < .001; *b* = -0.28, *SE* = .05, *p* < .001). At high (+1*SD*) levels of belief in the body as holy, such association was only marginally significant, *b* = -0.09, *SE* = .05, *p* = .059. However, there was not a statistically significant interaction between either religious construct and belief in body as sinful on IS (all *p*s > .231).

These moderation tests revealed that controlling for belief in body as holy changed the direction of the relationship between centrality of religion and IS from positive to negative. The change from positive to negative correlation indicates that the positive effect of centrality of religion on IS might be indirect, mediated through another variable (belief in body as holy). This led us to predict that belief in body as holy might actually mediate the relationship between centrality of religion and IS. Unlike moderation, where belief in body as holy influences the strength or direction of the centrality of religion and IS relationship, mediation suggests that centrality of religion influences belief in body as holy, which in turn affects IS. Theoretically, beliefs about the body represent plausible mechanisms (mediators) for the association between religiosity and IS. Religiosity may be associated with both beliefs but positively associated with IS *through* belief in body as holy–which is associated with more positive ways that people experience their bodies–and negatively *through* belief in body as sinful–which is associated with more negative ways that people experience their bodies [46].

**Fig 1:**
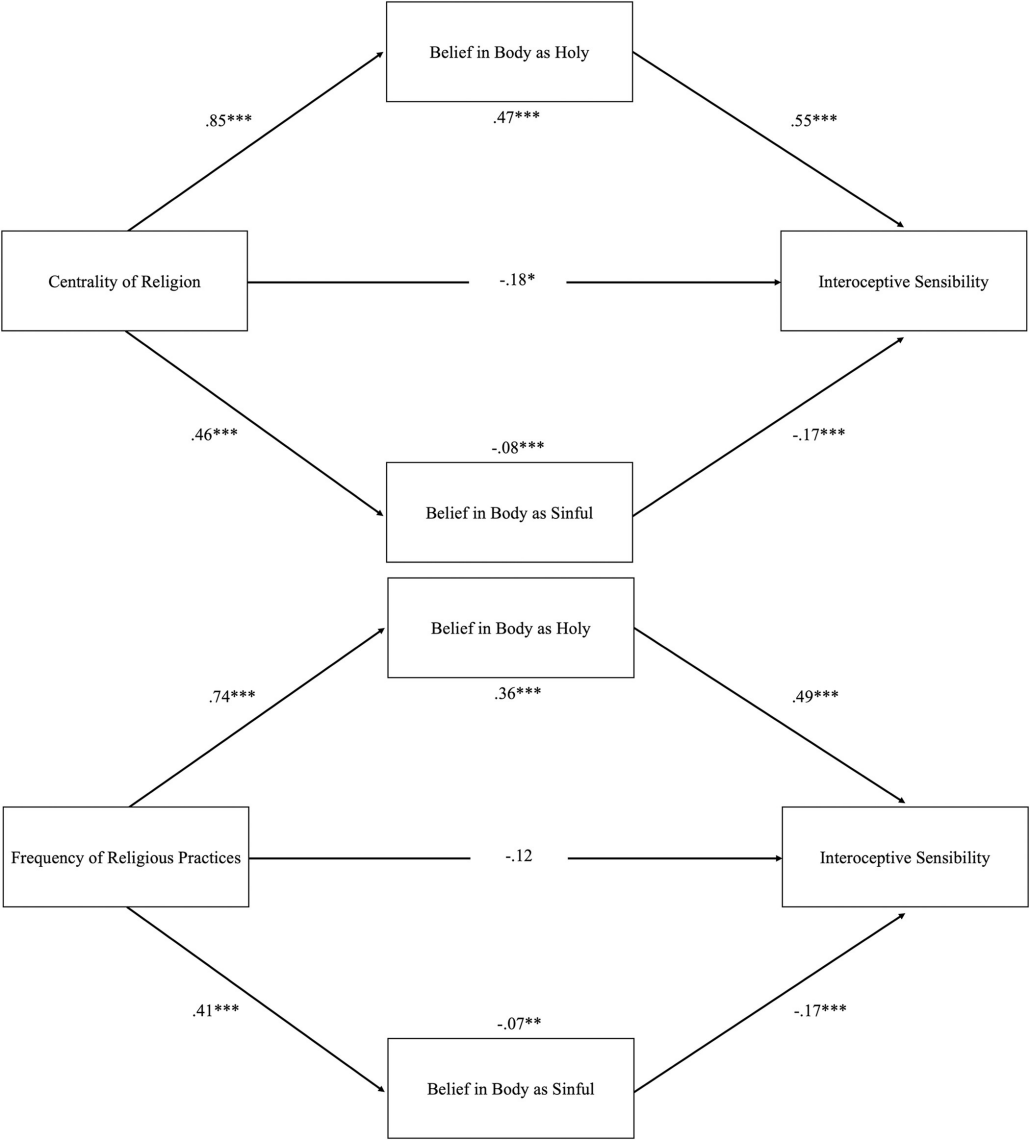
Beliefs About the Body as Mediators Between Centrality of Religion (top) or Frequency of Religious Practices (bottom) and IS. *Note*. Fully standardized coefficients are displayed. Top and bottom coefficients are for the indirect paths. * *p* < .05, ** *p* < .01, *** *p* < .001.

We explored whether beliefs about the body mediated the association between religiosity and IS. We tested for parallel mediation using the medmod module, GLM Mediation analysis in Jamovi. Regarding centrality of religion, the total indirect effect through belief in the body as holy was statistically significant, *B* = .47, *SE* = .02, *p* < .001, as well as the one through belief in the body as sinful, *B* = -0.08, *SE* = .01, *p* = .001. See Fig 1 for results for each path. Results show that while religiosity is positively associated with both types of beliefs about the body, belief in the body as holy is further positively associated (and partially explains religiosity’s association) with IS, whereas belief in the body as sinful is further negatively associated (and partially explains religiosity’s association) with IS. Descriptively, the mediation through belief in body as holy was stronger than belief in body as sinful. The same results emerged for frequency of religious practices. The total indirect effect through belief in the body as holy was statistically significant, *B* = 0.36, *SE* = .05, *p* < .001, as well as the one through belief in the body as sinful, *B* = -0.07, *SE* = .02, *p* = .001.

### IS dimension associations with beliefs about the body

Contrary to our expectations, the association between the Noticing subscale and belief in body as sinful was not confirmed (*r* = .02, *p* = .720). Surprisingly, this subscale is positively associated with belief in the body as holy (*r* = .19, *p* < .001). Attention Regulation was significantly positively associated with belief in the body as holy (*r* = .19, *p* < .001) as was Trusting (*r* = .27, *p* < .001) and the rest of the subscales included in the IS index, confirming our hypothesis. Our pre-registered hypotheses regarding the positive correlation between the Self-Regulation scale and belief in the body as holy was also confirmed (*r* = .34, *p* < .001), although the Self-Regulation scale was not included in the IS index.

We further note that the other two subscales that are not part of the IS index, due to measuring IS regulatory strategies as opposed to general IS, are significantly associated with belief in the body as sinful: Not Distracting (*r* = -.10, *p* = .040) and Not Worrying (*r* = -.11, *p* = .025). These two scales were the only ones not related to viewing the body as holy. Interestingly, both scales are often associated with maladaptive forms of interoception such as worrying about bodily sensations or ignoring important bodily signals [15]. These results could suggest those who either frequently worry about or ignore bodily signals more often view their bodies as bad or sinful.

## Discussion

This research sought to illuminate how religion, as a cultural factor, intertwines with perceptions and views of the body, an area that remains underexplored in current literature. In Studies 1 and 2, a positive association between religiosity and IS was found, which can be described as small to medium in size [57]. Notably, the association between how central religion is in one’s life and IS is larger among Muslim and Hindu participants compared to Christian participants (Study 1). Islam and Hinduism may involve more embodied practices or specific beliefs related to the body that might foster a heightened bodily awareness. These religious traditions are also more influenced by non-Western cultural norms. Interestingly, studies on cross-cultural differences in interoception and emotional concepts related to interoception, highlight the tendency for people from non-Western cultures to exhibit higher self-reported IS but lower interoceptive accuracy in comparison to Western cultures [26,61,62]. Indirect evidence suggests a greater cultural emphasis on bodily parts and processes among members of non-Western cultural groups—namely, East-Asians and West-Africans [26]. Future research may investigate more specifically the distinct or synergistic role of East-West culture and religion.

Interestingly, frequency of religious practices was not the strongest of the religious constructs to be associated with IS. Instead, the centrality of one’s religious identity and daily spiritual experiences were especially associated with IS. In the context of other research that has highlighted embodied contemplative practices as promoting IS [37], we wonder if spirituality is an important mechanism for the effects of such practices on IS [as it is for the effects of Mindfulness-Based Stress Reducation interventions on mental health, 63]. However, we also note that the present measure of religious practices may be too broad and not adequately capture the more specific practices in each religious tradition that may influence IS. Future research could also investigate how specific religious practices, such as prayer and fasting, directly influence IS. Investigating these practices across different religious traditions could offer valuable insights into the mechanisms through which religiosity impacts bodily awareness.

Study 2 delved deeper into how religious beliefs about the body play a role in shaping the relationship between religiosity and IS. The data suggested that the positive relationship between religiosity and IS is more accurately described as being partially mediated by beliefs about the body, rather than moderated by them. We found that religiosity is associated with both the belief in the body as sinful and as holy, but the links between these beliefs and IS differed. In line with research showing that IS is associated with more positive body image [49–51,61], belief in the body as holy partially explains the association between religiosity and IS, but belief in the body as sinful partially suppresses such association. Although body image may directly impact IS outside any religious influence, these results underscore the role that religion can play in shaping body image through specific religious-based views of the body, which ultimately could impact IS. Still, we highlight that such mediation results, exploratory and relying on cross-sectional data, should be interpreted with caution. The complex influence of religion, particularly specific religious doctrines, on how people perceive their ability to detect internal bodily states warrants future research.

### Limitations and future directions

One limitation of our study is the focus on specific religious traditions and views of the body, which may not be universally applicable across all faiths or cultural contexts. Both studies were also geographically limited to the U.S. Future research should explore a broader range of religious beliefs and practices, as well as their specific teachings related to the body, to further our understanding of how these elements interact with IS. Another limitation is the cross-sectional nature of the data collected. We cannot speak to any causality between religiosity and IS and causal language is only inferred theoretically.

Another limitation of this research is the reliance on self-reported scales. While the internal consistency of the MAIA is generally reliable when removing the Not-Worrying and Not-Distracting scales [18,19], the MAIA has faced criticism regarding its consistency and alignment with other IS questionnaires [64,65]. Future research should utilize multiple IS questionnaires such as the Body Awareness Questionnaire [66] and the Body Perception Questionnaire [67] to investigate factors that may be related to religiosity. Additionally, the use of self-report measures for interoception may introduce participant biases. Incorporating objective behavioral measures of interoception could provide further insight into the complex relationship between religion and body awareness, as these measures do not always align with self-reports and could reveal additional interoceptive processes, such as accuracy in detecting body signals. Furthermore, self-reported measures of religiosity, while validated and widely used, are susceptible to biases like social desirability. In particular, the selected items for religious-based beliefs about the body were drawn from longer published measures. Although selecting items for brevity is sometimes necessary, it can affect the internal consistency and the broad construct being measured.

Future research may investigate how IS affects other embodied processes in religious practices, such as the physical experience of practices around food intake or body purification, or the psychological experience of ritualistic postures and gestures [30,68]. Such research could examine how variations in IS might contribute to the intensity and quality of religious experiences, potentially influencing personal spirituality and communal religious engagement. This line of inquiry could also explore the reciprocal effects—how engaging in religious practices may, in turn, fine-tune IS, leading to a more integrated experience of religiosity that encompasses both mind and body.

Finally, perceptions of bodily signals are considered to be central to emotion experience in a range of theories of emotions [69–71]. Good aspects of IS have been associated with better emotion regulation [72] and may explain why religiosity has also been associated with more adaptive emotion regulation strategies [73].

## Conclusion

Recognizing the inherent benefits of IS–such as enhanced emotional regulation, improved stress management, and better mental health–our research provides preliminary data on the association between religious factors and body-based beliefs and awareness. We suggest that the relationship between multiple dimensions of religiosity and IS across religions is not only present, but is rich with implications for individual well-being, emotion regulation, and the broader practices of diverse faith communities. The integration of body-based awareness within religious contexts could provide new avenues for promoting mental health and emotional well-being within these communities. These areas are ripe for future research.

## Supporting information

S1 Table Full correlation matrix between IS, religious measures, and beliefs about the body (Study 2). (DOCX)

S2 Table Full correlation matrix between IS dimensions and religious measures for all and by religious group (Study 1). (DOCX)

S3 Table Full correlation matrix between IS dimensions, religious measures, and beliefs about the body (Study 2). (DOCX)

## References

[1] Wiens S. Interoception in emotional experience. Current Opinion in Neurology. 2005;18(4):442–7. doi: 10.1097/01.wco.0000168079.92106.99 16003122

[2] Hanley AW, Mehling WE, Garland EL. Holding the body in mind: Interoceptive awareness, dispositional mindfulness and psychological well-being. Journal of Psychosomatic Research. 2017;99:13–20. doi: 10.1016/j.jpsychores.2017.05.014 28712417 PMC5522814

[3] Farb N, Daubenmier J, Price CJ, Gard T, Kerr C, Dunn BD, et al. Interoception, contemplative practice, and health. Front Psychol. 2015;6:763. doi: 10.3389/fpsyg.2015.00763 26106345 PMC4460802

[4] Craig AD. How do you feel? Interoception: The sense of the physiological condition of the body. Nature Reviews Neuroscience. 2002;3(8):655–66. doi: 10.1038/nrn894 12154366

[5] Garfinkel SN, Seth AK, Barrett AB, Suzuki K, Critchley HD. Knowing your own heart: Distinguishing interoceptive accuracy from interoceptive awareness. Biological Psychology. 2015;104:65–74. doi: 10.1016/j.biopsycho.2014.11.004 25451381

[6] Khalsa SS, Adolphs R, Cameron OG, Critchley HD, Davenport PW, Feinstein JS, et al. Interoception and mental health: A roadmap. Biological Psychiatry: Cognitive Neuroscience and Neuroimaging. 2018;3(6):501–13. 10.1016/j.bpsc.2017.12.004 PMC6054486 29884281

[7] Legrand N, Nikolova N, Correa C, Brændholt M, Stuckert A, Kildahl N, et al. The heart rate discrimination task: A psychophysical method to estimate the accuracy and precision of interoceptive beliefs. Biological Psychology. 2022;168:108239. doi: 10.1016/j.biopsycho.2021.108239 34902450

[8] Kleckner IR, Wormwood JB, Simmons WK, Barrett LF, Quigley KS. Methodological recommendations for a heartbeat detection-based measure of interoceptive sensitivity. Psychophysiology. 2015;52(11):1432–40. doi: 10.1111/psyp.12503 26265009 PMC4821012

[9] Whitehead WE, Drescher VM, Heiman P, Blackwell B. Relation of heart rate control to heartbeat perception. Biofeedback and Self-regulation. 1977;2(4):371–92. 612350

[10] Suksasilp C, Garfinkel SN. Towards a comprehensive assessment of interoception in a multi-dimensional framework. Biological Psychology. 2022;168:108262. doi: 10.1016/j.biopsycho.2022.108262 35026353

[11] Schuette SA, Zucker NL, Smoski MJ. Do interoceptive accuracy and interoceptive sensibility predict emotion regulation? Psychological Research. 2021;85(5):1894–908. doi: 10.1007/s00426-020-01369-2 32556535

[12] Zamariola G, Vlemincx E, Corneille O, Luminet O. Relationship between interoceptive accuracy, interoceptive sensibility, and alexithymia. Personality and Individual Differences. 2018;125:14–20.

[13] Rimé B, Philippot P, Cisamolo D. Social schemata of peripheral changes in emotion. Journal of Personality and Social Psychology. 1990;59(1):38–49. doi: 10.1037//0022-3514.59.1.38 2213487

[14] Jones GE, Jones KR, Rouse CH, Scott DM, Caldwell JA. The effect of body position on the perception of cardiac sensations: An experiment and theoretical implications. Psychophysiology. 1987;24(3):300–11. doi: 10.1111/j.1469-8986.1987.tb00300.x 3602286

[15] Mehling WE, Price C, Daubenmier JJ, Acree M, Bartmess E, Stewart A. The Multidimensional Assessment of Interoceptive Awareness (MAIA). PloS One. 2012;7(11). doi: 10.1371/journal.pone.0048230 23133619 PMC3486814

[16] Mehling WE, Acree M, Stewart A, Silas J, Jones A. The Multidimensional Assessment of Interoceptive Awareness, Version 2 (MAIA-2). PLoS One. 2018;13(12). doi: 10.1371/journal.pone.0208034 30513087 PMC6279042

[17] Ventura-Bort C, Wendt J, Weymar M. The role of interoceptive sensibility and emotional conceptualization for the experience of emotions. Frontiers in Psychology. 2021;12. doi: 10.3389/fpsyg.2021.712418 34867591 PMC8636600

[18] Ferentzi E, Olaru G, Geiger M, Vig L, Köteles F, Wilhelm O. Examining the factor structure and validity of the Multidimensional Assessment of Interoceptive Awareness. J Pers Assess. 2021;103(5):675–84. doi: 10.1080/00223891.2020.1813147 32955947

[19] Da Costa Silva L, Belrose C, Trousselard M, Rea B, Seery E, Verdonk C, et al. Self-reported body awareness: Validation of the Postural Awareness Scale and the Multidimensional Assessment of Interoceptive Awareness (Version 2) in a non-clinical adult French-speaking sample. Frontiers in Psychology. 2022;13. 10.3389/fpsyg.2022.946271 PMC9362853 35959024

[20] Hanley AW, Mehling WE, Garland EL. Holding the body in mind: Interoceptive awareness, dispositional mindfulness and psychological well-being. J Psychosom Res. 2017;99:13–20. doi: 10.1016/j.jpsychores.2017.05.014 28712417 PMC5522814

[21] Barker E. Exploring the association between Interoceptive Awareness, Self-Compassion and Emotional Regulation [Unpublished Bachelor’s Honour Thesis]: University of Adelaide, Adelaide, Australia; 2019.

[22] Desdentado L, Miragall M, Rodríguez RL, Navarro MD, Baños RM. Identifying and regulating emotions after acquired brain injury: The role of interoceptive sensibility. Frontiers in Psychology. 2023;14. doi: 10.3389/fpsyg.2023.1268926 38179500 PMC10764614

[23] Eggart M, Todd J, Valdés-Stauber J. Validation of the Multidimensional Assessment of Interoceptive Awareness (MAIA-2) questionnaire in hospitalized patients with major depressive disorder. PLOS ONE. 2021;16(6). doi: 10.1371/journal.pone.0253913 34170963 PMC8232409

[24] Millon EM, Shors TJ. How mental health relates to everyday stress, rumination, trauma and interoception in women living with HIV: A factor analytic study. Learning and Motivation. 2021;73.

[25] Huang Y-H, Huang Y-T, Yen N-S. Interoceptive sensibility differentiates the predictive pattern of emotional reactivity on depression. Frontiers in Psychology. 2023;14. doi: 10.3389/fpsyg.2023.1011584 36936002 PMC10017445

[26] Ma-Kellams C. Cross-cultural differences in somatic awareness and interoceptive accuracy: A review of the literature and directions for future research. Frontiers in Psychology. 2014;5. doi: 10.3389/fpsyg.2014.01379 25520688 PMC4253951

[27] Saroglou V. Studying Religion in Personality and Social Psychology. In: Saroglou V, editor. Religion, personality, and social behavior. New York, NY: Psychology Press; 2014. p. 1–28.

[28] Pew Research Center. The changing global religious landscape 2017 [Available from: https://www.pewresearch.org/religion/2017/04/05/the-changing-global-religious-landscape/.

[29] Treves IN, Tello LY, Davidson RJ, Goldberg SB. The relationship between mindfulness and objective measures of body awareness: A meta-analysis. Scientific Reports. 2019;9(1):17386. doi: 10.1038/s41598-019-53978-6 31758073 PMC6874545

[30] Van Cappellen P, Edwards ME. The embodiment of worship: Relations among postural, psychological, and physiological aspects of religious practice. Journal for the Cognitive Science of Religion. 2021;6(1–2):56–79.

[31] Van Cappellen P, Edwards ME. Emotion expression in context: Full body postures of Christian prayer orientations compared to secular emotions. Journal of Nonverbal Behavior. 2021;45:545–65.

[32] Van Cappellen P, Edwards ME, Kamble S, Yildiz M, Ladd KL. Kneel, stand, prostrate: The psychology of prayer postures in three world religions. Plos One. 2024; 19(8). doi: 10.1371/journal.pone.0306924 39173058 PMC11341042

[33] Ring RM, Eisenmann C, Kandil FI, Steckhan N, Demmrich S, Klatte C, et al. Mental and Behavioural Responses to Bahá’í Fasting: Looking behind the Scenes of a Religiously Motivated Intermittent Fast Using a Mixed Methods Approach. Nutrients. 2022;14(5). 10.3390/nu14051038 PMC8912886 35268012

[34] Herbert BM, Herbert C, Pollatos O, Weimer K, Enck P, Sauer H, et al. Effects of short-term food deprivation on interoceptive awareness, feelings and autonomic cardiac activity. Biological Psychology. 2012;89(1):71–9. doi: 10.1016/j.biopsycho.2011.09.004 21958594

[35] Roeser RW. An introduction to Hindu India’s contemplative psychological perspective on motivation, self, and development. In: Maehr ML, Karabenick S, editors. Advances in Motivation and Achievement. Volume 14: Religion and Motivation. Amsterdam, The Netherlands: Elsevier; 2005. p. 297–345.

[36] Bornemann B, Herbert BM, Mehling WE, Singer T. Differential changes in self-reported aspects of interoceptive awareness through 3 months of contemplative training. Frontiers in Psychology. 2015;5. doi: 10.3389/fpsyg.2014.01504 25610410 PMC4284997

[37] Fischer D, Messner M, Pollatos O. Improvement of interoceptive processes after an 8-week body scan intervention. Frontiers in Human Neuroscience. 2017;11. 28955213 10.3389/fnhum.2017.00452 PMC5601051

[38] Mehling WE, Price C, Daubenmier J, Mike A, Bartmess E, Stewart A. Body awareness and the practice of yoga or mediation in 435 primary care patients with past or current low back pain. Journal of alternative and complementary medicine. 2014;20(5):A63–4.

[39] Khalsa SS, Rudrauf D, Damasio AR, Davidson RJ, Lutz A, Tranel D. Interoceptive awareness in experienced meditators. Psychophysiology. 2008;45(4):671–7. doi: 10.1111/j.1469-8986.2008.00666.x 18503485 PMC2637372

[40] Melloni M, Sedeño L, Couto B, Reynoso M, Gelormini C, Favaloro R, et al. Preliminary evidence about the effects of meditation on interoceptive sensitivity and social cognition. Behavioral and Brain Functions. 2013;9(1):47. doi: 10.1186/1744-9081-9-47 24365106 PMC3878404

[41] Saroglou V. Religion, spirituality, and altruism. Pargament JJE K. I., Jones J. W. editor: American Psychological Association; 2013.

[42] Voas D. Does religion belong in population studies? Environment and Planning A: Economy and Space. 2007;39(5):1166–80.

[43] Huber S, Huber OW. The centrality of religiosity scale (CRS). Religions. 2012;3(3):710–24.

[44] Pargament KI, Mahoney A. Spirituality: The search for the sacred. 2nd ed. Snyder SJL C. R., editor. New York: Oxford University Press; 2009.

[45] Hall MEL, Thoennes E. At home in our bodies: Implications of the incarnation for embodiment and Christian higher education. Christian Scholar’s Review. 2006;36:29–46.

[46] Jacobson HL, Hall MEL, Anderson TL, Willingham MM. Religious beliefs and experiences of the body: An extension of the developmental theory of embodiment. Mental Health, Religion & Culture. 2016;19(1):52–67.

[47] Mahoney A, Carels RA, Pargament KI, Wachholtz A, Leeper LE, Kaplar M, et al. The sanctification of the body and behavioral health patterns of college students. The International Journal for the Psychology of Religion. 2005;15(3):221–38.

[48] Badoud D, Tsakiris M. From the body’s viscera to the body’s image: Is there a link between interoception and body image concerns? Neuroscience and Biobehavioral Reviews. 2017;77:237–46. doi: 10.1016/j.neubiorev.2017.03.017 28377099

[49] Daubenmier JJ. The relationship of yoga, body awareness, and body responsiveness to self-objectification and disordered eating. Psychology of Women Quarterly. 2005;29(2):207–19.

[50] Todd J, Cardellicchio P, Swami V, Cardini F, Aspell JE. Weaker implicit interoception is associated with more negative body image: Evidence from gastric-alpha phase amplitude coupling and the heartbeat evoked potential. Cortex. 2021;143:254–66. doi: 10.1016/j.cortex.2021.07.006 34482968

[51] Todd J, Aspell JE, Barron D, Swami V. An exploration of the associations between facets of interoceptive awareness and body image in adolescents. Body Image. 2019;31:171–80. doi: 10.1016/j.bodyim.2019.10.004 31654981

[52] Ladd KL. Religiosity, the need for structure, death attitudes, and funeral preferences. Mental Health, Religion & Culture. 2007;10(5):451–72.

[53] Ladd KL, Spilka B. Inward, outward, upward prayer: Scale reliability and validation. Journal for the Scientific Study of Religion. 2006;45(2):233–51.

[54] Self-schemata Markus H. and processing information about the self. Journal of Personality and Social Psychology. 1977;35(2):63–78.

[55] Idler EL, Musick MA, Ellison CG, George LK, Krause N, Ory MG, et al. Measuring multiple dimensions of religion and spirituality for health research conceptual background and findings from the 1998 General Social Survey. Research on Aging. 2003;25(4):327–65.

[56] Eisinga R, Grotenhuis Mt, Pelzer B. The reliability of a two-item scale: Pearson, Cronbach, or Spearman-Brown? International Journal of Public Health. 2013;58(4):637–42. 23089674 10.1007/s00038-012-0416-3

[57] Gignac GE, Szodorai ET. Effect size guidelines for individual differences researchers. Personality and Individual Differences. 2016;102:74–8.

[58] Saroglou V, Clobert M, Cohen AB, Johnson KA, Ladd KL, Van Pachterbeke M, et al. Believing, bonding, behaving, and belonging: The cognitive, emotional, moral, and social dimensions of religiousness across cultures. Journal of Cross-Cultural Psychology. 2020;51(7–8):551–75.

[59] Underwood LG, Teresi JA. The daily spiritual experience scale: Development, theoretical description, reliability, exploratory factor analysis, and preliminary construct validity using health-related data. Annals of Behavioral Medicine. 2002;24(1):22–33. doi: 10.1207/S15324796ABM2401_04 12008791

[60] Saroglou V, Munoz-Garcia A. Individual differences in religion and spirituality: An issue of personality traits and/or values. Journal for the Scientific Study of Religion. 2008;47:83–101.

[61] Chentsova-Dutton YE, Dzokoto V. Listen to your heart: The cultural shaping of interoceptive awareness and accuracy. Emotion. 2014;14(4):666–78. doi: 10.1037/a0036193 24749640

[62] Zhou P, Critchley H, Garfinkel S, Gao Y. The conceptualization of emotions across cultures: A model based on interoceptive neuroscience. Neuroscience & Biobehavioral Reviews. 2021;125:314–27. 10.1016/j.neubiorev.2021.02.023 33631316

[63] Greeson JM, Webber DM, Smoski MJ, Brantley JG, Ekblad AG, Suarez EC, et al. Changes in spirituality partly explain health-related quality of life outcomes after mindfulness-based stress reduction. Journal of Behavioral Medicine. 2011;34(6):508–18. doi: 10.1007/s10865-011-9332-x 21360283 PMC3151546

[64] Vig L, Köteles F, Ferentzi E. Questionnaires of interoception do not assess the same construct. PLoS ONE 2022;17(8). 10.1371/journal.pone.0273299 PMC9397851 35998182

[65] Desmedt O, Heeren A, Corneille O, Luminet O. What do measures of self-report interoception measure? Insights from a systematic review, latent factor analysis, and network approach. Biological Psychology. 2022;169:108289. doi: 10.1016/j.biopsycho.2022.108289 35150768

[66] Shields SA, Mallory ME, Simon A. The Body Awareness Questionnaire: Reliability and validity. Journal of Personality Assessment. 1989;53(4):802–15.

[67] Porges S. Body Perception Questionnaire. College Park, MA: University of Maryland. 1993.

[68] Van Cappellen P, Cassidy S, Zhang R. Religion as an embodied practice: Documenting the various forms, means, and associated experience of Christian prayer postures. Psychology of Religion and Spirituality. 2023;15:251–61.

[69] James W. What is an emotion? Mind. 1884;34:188–205.

[70] Barrett LF, Lindquist K. The embodiment of emotion. In: Semin G, Smith E, editors. Embodied grounding: Social, cognitive, affective, and neuroscience approaches. New York, NY: Cambridge University Press; 2008. p. 237–62.

[71] Damasio AR. Descartes’ error: Emotion, Reason and the Human Brain. New York, NY: Grosset/Putnam; 2006.

[72] Zamariola G, Frost N, Van Oost A, Corneille O, Luminet O. Relationship between interoception and emotion regulation: New evidence from mixed methods. Journal of Affective Disorders. 2019;246:480–5. doi: 10.1016/j.jad.2018.12.101 30599372

[73] Vishkin A, Ben-Nun Bloom P, Schwartz SH, Solak N, Tamir M. Religiosity and emotion regulation. Journal of Cross-Cultural Psychology. 2019;50(9):1050–74.

