# Supplementary material for: Bodily awareness: Religious culture’s associations with interoceptive sensibility
Source: PLoS One. 2024 Dec 2;19(12):e0309216. doi: 10.1371/journal.pone.0309216 (PMC11611216; doi:10.1371/journal.pone.0309216)
Supplement: S1 Table — (DOCX) [file pone.0309216.s001.docx]

**Supporting Information**

**S1 Table. Study 1 and 2 means and standard deviations (Including IS dimensions).**

|  | Study 1 | | | | | | | | Study 2 | |
| --- | --- | --- | --- | --- | --- | --- | --- | --- | --- | --- |
|  | All religions | | Christians | | Muslims | | Hindus | |  | |
|  | *M* | *SD* | *M* | *SD* | *M* | *SD* | *M* | *SD* | *M* | *SD* |
| IS | 3.37 | .67 | 3.33 | .73 | 3.42 | .65 | 3.34 | .56 | 3.30 | .84 |
| Noticing | 3.13 | .88 | 3.24 | .91 | 3.08 | .89 | 3.00 | .81 | 3.53 | 1.01 |
| Trusting | 3.69 | .83 | 3.71 | .89 | 3.70 | .81 | 3.63 | .75 | 3.55 | 1.15 |
| Attention  Regulation | 3.37 | .76 | 3.25 | .85 | 3.50 | .71 | 3.42 | .65 | 3.21 | .95 |
| Body  Listening | 3.02 | .91 | 2.83 | 1.00 | 3.20 | .84 | 3.09 | .77 | 2.86 | 1.28 |
| Emotional  Awareness | 3.62 | .78 | 3.64 | .86 | 3.64 | .74 | 3.58 | .69 | 3.35 | 1.07 |
| Self-  Regulation | N/A | N/A | N/A | N/A | N/A | N/A | N/A | N/A | 3.06 | 1.16 |
| Not-  Distracting | N/A | N/A | N/A | N/A | N/A | N/A | N/A | N/A | 3.11 | 1.11 |
| Not-  Worrying | N/A | N/A | N/A | N/A | N/A | N/A | N/A | N/A | 3.24 | 1.03 |
| Religious Centrality | 4.41 | 1.28 | 4.24 | 1.42 | 4.57 | 1.20 | 4.49 | 1.09 | 3.75 | 3.11 |
| Frequency of Religious Practice (z-score) | 0 | .82 | -.23 | .88 | .27 | .73 | .05 | .69 | 0 | .89 |
| 4 Dimensions of Religiousness | N/A | N/A | N/A | N/A | N/A | N/A | N/A | N/A | 3.04 | 2.00 |
| Belonging | N/A | N/A | N/A | N/A | N/A | N/A | N/A | N/A | 2.84 | 2.07 |
| Behaving | N/A | N/A | N/A | N/A | N/A | N/A | N/A | N/A | 2.99 | 2.23 |
| Believing | N/A | N/A | N/A | N/A | N/A | N/A | N/A | N/A | 3.16 | 2.04 |
| Bonding | N/A | N/A | N/A | N/A | N/A | N/A | N/A | N/A | 3.15 | 1.97 |
| Importance of Spirituality | N/A | N/A | N/A | N/A | N/A | N/A | N/A | N/A | 4.43 | 3.21 |
| Daily Spiritual Experiences | N/A | N/A | N/A | N/A | N/A | N/A | N/A | N/A | 2.68 | 1.64 |
| Body As Holy | N/A | N/A | N/A | N/A | N/A | N/A | N/A | N/A | 3.34 | 2.25 |
| Body As Sinful | N/A | N/A | N/A | N/A | N/A | N/A | N/A | N/A | 2.16 | 1.70 |
