# Supplementary material for: Bodily awareness: Religious culture’s associations with interoceptive sensibility
Source: PLoS One. 2024 Dec 2;19(12):e0309216. doi: 10.1371/journal.pone.0309216 (PMC11611216; doi:10.1371/journal.pone.0309216)
Supplement: S2 Table — (DOCX) [file pone.0309216.s002.docx]

**S2 Table. Full correlation matrix between IS dimensions and religious measures for all and by religious group (Study 1).**

|  | All | | Christians | | Muslims | | Hindus | |
| --- | --- | --- | --- | --- | --- | --- | --- | --- |
|  | Centrality of Religion | Frequency of Religious Practices | Centrality of Religion | Frequency of Religious Practices | Centrality of Religion | Frequency of Religious Practices | Centrality of Religion | Frequency of Religious Practices |
| IS | .30*** | .17*** | .20*** | .13*** | .37*** | .17*** | .48*** | .23*** |
| Noticing | .19*** | .06* | .16*** | .05 | .24*** | .10* | .27*** | .15** |
| Trusting | .28*** | .13*** | .19** | .14*** | .36*** | .12** | .42*** | .15** |
| Attention  Regulation | .28*** | .18*** | .18*** | .11** | .34*** | .17*** | .41*** | .25*** |
| Body  Listening | .25*** | .18*** | .15*** | .12** | .28*** | .16** | .39*** | .18*** |
| Emotional  Awareness | .25*** | .12*** | .17*** | .11** | .31*** | .13** | .42*** | .17*** |
