# Supplementary material for: Bodily awareness: Religious culture’s associations with interoceptive sensibility
Source: PLoS One. 2024 Dec 2;19(12):e0309216. doi: 10.1371/journal.pone.0309216 (PMC11611216; doi:10.1371/journal.pone.0309216)
Supplement: S3 Table — (DOCX) [file pone.0309216.s003.docx]

**S3 Table. Full correlation matrix between IS dimensions, religious measures, and beliefs about the body (Study 2)**

|  | **1** | **2** | **3** | **4** | **5** | **6** | **7** | **8** | **9** | **10** | **11** | **12** | **13** | **14** | **15** | **16** | **17** | **18** | **19** |
| --- | --- | --- | --- | --- | --- | --- | --- | --- | --- | --- | --- | --- | --- | --- | --- | --- | --- | --- | --- |
| 1. IS | — |  |  |  |  |  |  |  |  |  |  |  |  |  |  |  |  |  |  |
| 2. Noticing | .72*** | — |  |  |  |  |  |  |  |  |  |  |  |  |  |  |  |  |  |
| 3. Trusting | .68*** | .24*** | — |  |  |  |  |  |  |  |  |  |  |  |  |  |  |  |  |
| 4. Attention  Regulation | .85*** | .49*** | .61*** | — |  |  |  |  |  |  |  |  |  |  |  |  |  |  |  |
| 5. Body  Listening | .79*** | .49*** | .49*** | .51*** | — |  |  |  |  |  |  |  |  |  |  |  |  |  |  |
| 6. Emotional  Awareness | .83*** | .60*** | .38*** | .53*** | .67*** | — |  |  |  |  |  |  |  |  |  |  |  |  |  |
| 7. Self-  Regulation | .74*** | .36*** | .61*** | .62*** | .67*** | .60*** | — |  |  |  |  |  |  |  |  |  |  |  |  |
| 8. Not  Distracting | .05 | .01 | .15** | .03 | .07 | -.03 | .04 | — |  |  |  |  |  |  |  |  |  |  |  |
| 9. Not  Worrying | .16*** | -.05 | .32*** | .32*** | .03 | -.05 | .26*** | 0 | — |  |  |  |  |  |  |  |  |  |  |
| 10. General Religiosity | .21*** | .11* | .19*** | .11* | .23*** | .22*** | .24*** | -.06 | .01 | — |  |  |  |  |  |  |  |  |  |
| 11. Frequency of Religious Practice | .17*** | .09 | .15** | .10* | .18*** | .18*** | .21*** | -.03 | .04 | .83*** | — |  |  |  |  |  |  |  |  |
| 12. Dimensions of Religiousness | .22*** | .10* | .18*** | .14** | .23*** | .24*** | .25*** | -.07 | .03 | .90*** | .81*** | — |  |  |  |  |  |  |  |
| 13. Belonging | .22*** | .10* | .17*** | .14** | .23*** | .22*** | .23*** | -.07 | .04 | .87*** | .79*** | .97*** | — |  |  |  |  |  |  |
| 14. Behaving | .20*** | .09 | .16*** | .12** | .20*** | .21*** | .22*** | -.06 | .03 | .89*** | .79*** | .97*** | .93*** | — |  |  |  |  |  |
| 15. Believing | .21*** | .08 | .17*** | .12** | .24*** | .23*** | .25*** | -.06 | .01 | .89*** | .79*** | .97*** | .91*** | .93*** | — |  |  |  |  |
| 16. Bonding | .24*** | .12* | .19*** | .17*** | .22*** | .25*** | .25*** | -.09 | .04 | .80*** | .72*** | .94*** | .90*** | .86*** | .88*** | — |  |  |  |
| 17. Importance of Spirituality | .26*** | .15** | .21*** | .15** | .27*** | .28*** | .29*** | -.03 | 0 | .84*** | .69*** | .76*** | .73*** | .73*** | .78*** | .69*** | — |  |  |
| 18. Daily Spiritual Experience | .32*** | .21*** | .26*** | .21*** | .30*** | .31*** | .32*** | -.06 | .05 | .90*** | .82*** | .85*** | .81*** | .83*** | .85*** | .77*** | .83*** | — |  |
| 19. Body as Holy | .33*** | .19*** | .28*** | .20*** | .32*** | .31*** | .34*** | -.06 | .04 | .85*** | .74*** | .78*** | .74*** | .76*** | .80*** | .70*** | .81*** | .88*** | — |
| 20. Body as Sinful | .01 | .02 | .01 | -.07 | .04 | .06 | .09 | -.10* | -.11* | .45*** | .41*** | .39*** | .38*** | .39*** | .38*** | .35*** | .38*** | .40*** | .45*** |

Note. Analyses control for Condition variable. * p < .05, ** p < .01, *** p < .001
